# Molecular Machinery and Pathophysiology of Mitochondrial Dynamics

**DOI:** 10.3389/fcell.2021.743892

**Published:** 2021-09-17

**Authors:** Yi-Han Chiu, Shu-Chuan Amy Lin, Chen-Hsin Kuo, Chia-Jung Li

**Affiliations:** ^1^Department of Microbiology, Soochow University, Taipei, Taiwan; ^2^Department of Nursing, National Yang Ming Chiao Tung University Hospital, Yilan, Taiwan; ^3^School of Nursing, National Yang Ming Chiao Tung University, Taipei, Taiwan; ^4^Department of Obstetrics and Gynecology, Kaohsiung Veterans General Hospital, Kaohsiung, Taiwan; ^5^Institute of BioPharmaceutical Sciences, National Sun Yat-sen University, Kaohsiung, Taiwan

**Keywords:** mitochondrial dynamics, fusion, fission, pathophysiology, machinery

## Abstract

Mitochondria are double-membraned organelles that exhibit fluidity. They are the main site of cellular aerobic respiration, providing energy for cell proliferation, migration, and survival; hence, they are called “powerhouses.” Mitochondria play an important role in biological processes such as cell death, cell senescence, autophagy, lipid synthesis, calcium homeostasis, and iron balance. Fission and fusion are active processes that require many specialized proteins, including mechanical enzymes that physically alter mitochondrial membranes, and interface proteins that regulate the interaction of these mechanical proteins with organelles. This review discusses the molecular mechanisms of mitochondrial fusion, fission, and physiopathology, emphasizing the biological significance of mitochondrial morphology and dynamics. In particular, the regulatory mechanisms of mitochondria-related genes and proteins in animal cells are discussed, as well as research trends in mitochondrial dynamics, providing a theoretical reference for future mitochondrial research.

## Introduction

Mitochondria are organelles composed of an outer mitochondrial membrane (OMM) and an inner mitochondrial membrane (IMM), forming an inter-membrane space (IM) between. Reactions in the mitochondrial matrix provide energy for the normal functions of different cell types. Under physiological conditions, the OMM and IMM work together, continuously undergoing fusion and fission. While mitochondrial fusion and fission are essential for cellular homeostasis (Wu et al., 2018) and mitochondrial function, each physiological process plays a different role in mitochondrial function. Mitochondrial fusion can lead to an extension of mitochondrial structure, an increase in ATP content, and the transfer of various mitochondrial active substances to the newly fused mitochondria (Li et al., 2017). When cells are subjected to stress conditions (e.g., starvation or light stimulation), mitochondrial fusion reaches a maximum, producing enough ATP to counteract the stress (Silva Ramos et al., 2016). In contrast to fusion, mitochondrial fission produces new, smaller mitochondria that contribute to cell fission and mitosis. In addition, mitochondrial division, a process of mitochondrial proliferation, is ongoing and highly co-ordinated in eukaryotic cells and can also help maintain cellular constancy through mitophagy (Twig et al., 2008). Mitochondrial biogenesis and autophagy are highly regulated by fusion or fission, which also highlights the enormous impact of mitochondrial homeostasis on cellular function. As shown in Table 1, when mitochondrial fusion and/or fission are damaged, mitochondrial function is disrupted, which can eventually lead to neurodegenerative diseases, cardiovascular diseases, metabolic diseases, as well as many others (Archer, 2013, 2014).

**TABLE 1:** Summary of mitochondrial morphological effect proteins.

**Protein**	**Effects**	**References**
Mfn1	It is a transmembrane GTPase and mediates mitochondrial fusion. Mitochondrial fusion occurs in a variety of cell types and is an important step in the balance between fusion and fission.	Civiletto et al., 2015
Mfn2	It is a transmembrane GTPase and mediates mitochondrial fusion. Mitochondrial fusion occurs in a variety of cell types and is an important step in the balance between fusion and fission.	Civiletto et al., 2015
Opa1	It is a GTPase associated with mitochondrial fusion and apoptosis. Its formation is used to store proteins within the mitochondrial cristae to prevent proliferation.	Gomes et al., 2011
L-Opa1	The Opa1 is a dynamin-related protein associated with the inner mitochondrial membrane and functions in mitochondrial inner membrane fusion and cristae maintenance.	Gonzalez-Franquesa and Patti, 2017
S-Opa1	Inner membrane-anchored L-Opa1 undergoes proteolytic cleavage resulting in S-Opa1.	Gonzalez-Franquesa and Patti, 2017
Drp1	It mainly compresses around the break site by the mechanism of hydrolysis of GTP and then cuts off the mitochondrial membrane.	Huttemann et al., 2011
Fis1	It is involved in fragmentation fission and perinuclear clustering factors of the mitochondrial reticular organization.	Ishihara et al., 2006
Mff	Promotion of cleavage-mediated Drp1 to the mitochondrial surface is associated with recruitment	Kaltenbach et al., 2007
Usp30	Deubiquitinating enzymes tethered to the OMM act as key inhibitors of autophagy by counteracting the action of parkin.	Kamerkar et al., 2018
Mtp18	Involved in mitochondrial fission through regulation of membrane fission.	Kim et al., 2011
MitoPLD	It is located in the OMM and regulates mitochondrial dynamics.	Korobova et al., 2013
Oma1	Part of the quality control system of the IMM regulates the depolarization of the ΔΨ*m*.	Koshiba et al., 2004
Yme1l	Maintains mitochondrial morphology and complex respiratory activity.	Lackner, 2013
Mid49	GTPase activity in OMM proteins that control mitochondrial fission and regulate Drp1.	Leduc-Gaudet et al., 2021
Mid51	GTPase activity in OMM proteins that control mitochondrial fission and regulate Drp1.	Lee et al., 2016

## Overview of Mitochondrial Fusion and Fission

### Physiological Significance of Mitochondrial Dynamic Homeostasis

The structure of mitochondria and the synthesis of particular products are influenced by the needs of the cells in which they are located. Both the structure and metabolism of mitochondria play a key role in cell cycle progression, cell differentiation, development, the immune response, dynamic regulation of lipids and calcium, and apoptosis, in addition to influencing the generation of energy required by the cell (Nunnari and Suomalainen, 2012). Dynamin-related proteins (DRPs) mediate dynamic fusion and fission of mitochondria and remodel membrane shape, mainly through GTP-dependent self-assembly and GTP hydrolysis-mediated conformational changes (Lackner, 2013). Among different species, dynamin-1 protein (Dnm1, yeast) and dynamin-related protein 1 (Drp1, mammals) similarly lead to mitochondrial membrane fission, and mitofusins [Fzo1 (yeast)/Mfn1 and Mfn2 (mammals)] and the DRP Mgm1 (yeast)/Opa1 (mammals) mediate the fusion of outer the OMM and inner mitochondrial membranes the IMM, respectively (Gao and Hu, 2021).

**FIGURE 1:**
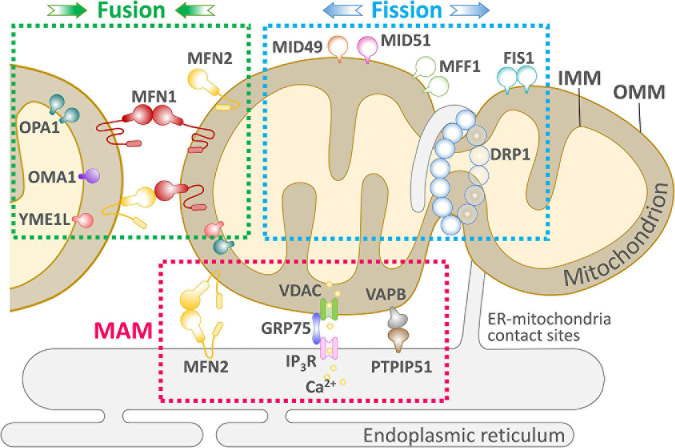
An overview of the key components of mitochondrial dynamics-ER communication and maintenance of cellular physiology. Mitochondria undergo two successive and opposite processes related to membranes: fission and fusion. Depending on the cellular environment and circumstances, the balance between fission and fusion can change, and the balance tends to favor one of these two processes. When cells are subjected to mild stress, mitochondria form an elongated and interconnected network to adapt to nutrient deficiencies while adapting to cellular stress. In contrast, under severe stress, mitochondria present a fragmented shape. Mitochondria and ER are in close proximity to each other and form the MAM. The MAM allows efficient Ca^2+^-stable exchange between the ER and mitochondria through the IP_3_R, VDAC mitochondrial calcium unidirectional transport protein (MCU) and PTP. Several proteins involved in mitochondrial fission, such as Mff and Fis1 mediate the localization of Drp1 on the ER, suggesting the existence of other unknown pathways to control ER-controlled mitochondrial dynamics.

Rapid mitochondrial fusion and fission are mechanisms for eliminating abnormal mitochondria from the cell (Yu et al., 2020). Mitochondrial fusion and fission are paired in a continuous progression, and cumulative probability analysis indicates that fusion triggers fission, but fission cannot affect the process of fusion that occurs later. Moreover, mitochondrial fusion is selective; after fission, depolarized daughter mitochondria are unlikely to participate in later fusions and will eventually be eliminated by autophagy (Chen Z. et al., 2018). As shown in Figure 1, mitochondrial fusion is mediated by the interaction of Mfn1 and Mfn2 at the OMM and Opa1 at the IMM; Fis1, Mff, and Mid49/51 drive the receptor-mediated enrichment of Drp1 from the cytoplasm to the OMM to the endoplasmic reticulum (ER)-labeled fission site, driving mitochondrial fission (Wai and Langer, 2016).

Mitochondria change their morphological structure (elongating, shortening, bifurcating, bending, and swelling) by fusion and fission, maintaining the physiological functions of the cell. Mitochondrial fusion is mainly involved in the synthesis of new mitochondria and the repair of damaged mitochondria [e.g., mitochondrial DNA (mtDNA) mutations, decrease in membrane potential]. When damaged mitochondria fuse with normal mitochondria, mtDNA is reintegrated, repaired, and the membrane potential adjusts to normal levels (Gaziev et al., 2014). When an organism is under stress (e.g., disease, starvation, etc.), mitochondrial fusion leads to maximal ATP production, which supports the energy needs of the organism (Silva Ramos et al., 2016). Mitochondrial fusions include OMM fusions and IMM fusions. OMM fusions are mainly mediated by functionally similar Mfn1 and Mfn2 proteins (Table 1; Sloat et al., 2019).

### Outer Membrane Fusion-Associated Proteins

Homologous mammalian genes (Mfn1 and Mfn2) regulate the fusion of the OMM and maintain the dynamic needs of reticulate mitochondria in the cell (Mishra and Chan, 2014). Both Mfn1 and Mfn2 are OMM transmembrane GTPases containing several conserved regions, including the amino-terminal GTP-binding structural domain, two coiled-coil structural domains, and the carboxy-terminus of a transmembrane structural domain (Chandhok et al., 2018). They are anchored to the OMM via a C-terminal transmembrane structural domain and mediate OMM fusion through homo- and heterotypic interactions with GTP hydrolysis. The impairment of either Mfn2 or GTPase activity prevents normal mitochondrial fusion, resulting in a small mitochondrial network in cells that fracture during mitochondrial fission (Escobar-Henriques and Joaquim, 2019).

Although Mfn1 and Mfn2 have similar functions and are even functionally complementary in specific situations, only mutations in Mfn2 cause significant physiological changes leading to neurodegenerative diseases, such as type 2A peroneal muscular atrophy neuropathy (Sloat et al., 2019). The two proteins play different functions in mitochondrial fusion, e.g., mitochondrial elongation under hypoxia is mainly regulated by sirt1-mediated Mfn1 deacetylation (Oanh et al., 2017), whereas Mfn1 mediates the formation of mitochondrial and ER contacts (Basso et al., 2018). The difference in functions of Mfn1 and Mfn2 may be due to the lack of the N-terminal RAS binding domain in Mfn1 (Chen et al., 2004). The membrane proteins responsible for mitochondrial fusion mediate fusion on adjacent mitochondria, indicating that the fusion complex needs to be between adjacent mitochondria to function. That is, the second coiled-coil domain of each protein forms an anti-parallel coiled-coil structure after forming a homo- or heterodimer, thereby restraining and regulating mitochondrial fusion. Therefore, mitochondrial fusion proteins promote fusion mainly through the action of molecular dimers on neighboring mitochondria (Koshiba et al., 2004).

### Inner Membrane Fusion-Associated Proteins

Two forms of Opa1 have been identified in mammalian cells and tissues as a result of selective splicing and proteolytic cleavage by two endosomal peptidases: Oma1 and the i-AAA protease Yme1l. The Opa1 form obtained by selective splicing is the long-chain Opa1 (L-Opa1), whereas proteolytic cleavage generates the short-chain Opa1 (S-Opa1). L-Opa1 is anchored to the IMM through the N-terminal transmembrane structural domain and is released by proteolytic cleavage, producing the soluble form of S-Opa1 (Ishihara et al., 2006). Thus, L-Opa1 processing helps maintain the balance between mitochondrial fusion and cleavage, an important regulatory mechanism of the mitochondrial network structure (Anand et al., 2013). In contrast, IMM fusion is mainly mediated by Opa1, which is responsible for joining and integrating the two IMM systems and forming a complete IMM system when the OMM is fused (Figure 1). In mitochondria, Opa1 is proteolytically cleaved into two isoforms, long and short, but the two proteins alone are not physiologically active and only interact while mediating mitochondrial fusion (Del Dotto et al., 2018). The relative concentrations of the two isoforms are nearly equal in the cell, which is necessary for proper function in IMM fusion in normal cellular physiology (Sanchis-Gomar and Derbre, 2014). Mitochondrial fusion is closely related to cellular physiological function, and fusion transfers mitochondrial proteins and mtDNA to newly synthesized mitochondria, which helps prevent the accumulation of damaged mtDNA (Chan, 2020). In contrast, the rate of mitochondrial fusion can be affected by the physicochemical properties of mitochondria, such as changes in mitochondrial membrane potential. In a study of pancreatic cancer cells in G1 phase with high/low metabolism to alter the cellular mitochondrial membrane potential, the mitochondrial membrane potential increased; oxidative phosphorylation (OxPhos) as well as mitochondrial respiration and fusion rates were correspondingly elevated (Little et al., 2020).

### Mitochondrial Fission-Related Proteins

Mitochondrial fission plays a balancing role with mitochondrial fusion in mitochondrial dynamics. There are two main modes of mitochondrial fission: (1) “Inside-out,” in which the IMM fractures first, eventually leading to fracture of the OMM, and (2) “Extrusion,” in which the mitochondria accumulate from the fission point inward, leading to mitochondrial fission. Currently, most of the proteins known to be associated with mitochondrial fission are GTPase family proteins, including Drp1, Fis1, Mff, and Mid49/51 (Chen K. H. et al., 2018; Kamerkar et al., 2018; Yu et al., 2019a,b). Also present are dynamin protein 2 (Dyn2/Dnm2), and Bax interacting factor 1 (Bif1), which assist Drp1 in regulating mitochondrial fission (Mahecic et al., 2021), and ganglioside-induced differentiation-associated protein 1 (GDAP1) (Rzepnikowska and Kochanski, 2018), death-associated protein 3 (DAP3) (Xiao et al., 2015), and mitochondria protein 18 (MTP18) (Aung et al., 2019). Maintaining physiological constancy consumes energy, and the energy required for mitochondrial fission is mainly provided by the hydrolysis of GTP by GTPases. However, the fission process requires the involvement of the ER, which helps initiate mitochondrial fission before GTPase hydrolysis. As shown in Figure 1, mitochondrial fission begins with “tagging” of the mitochondrial membrane by the ER and the ER-mitochondrial contact sites (ERMCSs), resulting in the recruitment of Drp1 by the Drp1 receptor. Drp1 then forms oligomers around the fission site, further constricting membrane GTPase activity, tightening the mitochondria, and leading to Dyn1/Dyn2 replenishment, while allowing additional GTPase activity. Dyn2 replenishment allows for additional GTP hydrolysis to complete the cleavage process, producing two separate mitochondria. Mitochondrial fission is essential for intracellular mitochondrial remodeling and rearrangement, and for the transfer of healthy mtDNA and other active material to daughter cells after mitotic fission (Pagliuso et al., 2018). Drp1 performs OMM fission, mediating mitochondrial fission, and in response to specific cellular signals, Drp1 moves from the cytoplasm to the OMM, where it clusters into a loop at the fission site. Several Drp1 receptor recruitment elements at the OMM have been identified, including Fis1, Mff, Mid49, and Mid51 (Civiletto et al., 2015). The mechanism of inner membrane fission is still unclear. Two IMM proteins, S-Opa1 and MTP18, have been suggested to have an important role in mitochondrial fission and may be part of the endosomal fission mechanism (Wai and Langer, 2016).

## Regulation of Mitochondrial Fission and ER Connection

### Role of ER-Mitochondrial Contact Sites

Contact sites exist between mitochondria and the ER and these sites are critical for phospholipid synthesis, Ca^2+^ homeostasis, and labeling of cleavage sites (Friedman et al., 2011). In mammals, the function of the contact sites is mainly regulated by Mfn2 (de Brito and Scorrano, 2008). Although mitochondria are dynamic, the location of mitochondrial and ER contact sites is kept relatively constant; the exact mechanism of regulation needs to be further investigated (Friedman et al., 2010). Many researchers have suggested that the role of the ER is to mark the initiation site of mitochondrial fusion and fission (Friedman et al., 2011). IFN2 located in the ER induces the polymerization of actin at the interface between the ER and mitochondrial contact sites, driving contraction of mitochondrial fission sites, probably because Drp1 oligomers cannot wrap the mitochondrial membrane and induce fission in the absence of receptors (Korobova et al., 2013). Depending on the cell type, mitochondria are generally greater than 200 nm in diameter, and the Drp1 oligomers recognize and bind mitochondrial membranes with diameters of 110–130 nm before further contraction of the membrane structure. In contrast, ER contraction sites are approximately 138–146 nm in diameter, suggesting that ER-initiated contraction precedes the formation of Drp1 oligomers at mitochondrial membrane contraction sites (Friedman et al., 2011). This suggests that the ER plays an important role in initiating contraction prior to mitochondrial contraction, and it also demonstrates that the ER is the initiation site for labeling mitochondrial contraction and fission (Korobova et al., 2013).

### Mitochondrial Fission Protein Regulatory Mechanisms

Mitochondrial fission is mainly regulated by Drp1, Fis1, Dyn1/2, Mff, and other related genes and proteins. Drp1 is primarily localized to the cytoplasmic matrix, but Drp1 does not possess a lipid-binding pleckstrin homology domain and cannot bind directly to the mitochondrial membrane. Therefore, its mediation of mitochondrial fission requires recruitment to the mitochondria (Mears et al., 2011). As shown in Figure 1, mitochondrial proteins must act as Drp1 receptors to cluster Drp1 into the OMM. In mammalian cells, Drp1 receptors identified include Fis1, Mff, and Mid49/51 (Otera et al., 2010; Palmer et al., 2011; Zhao et al., 2011; Loson et al., 2013); all three can recruit Drp1 to the OMM with Mff having the strongest binding effect (Loson et al., 2013). Once Drp1 is recruited to the OMM, it forms a cyclic oligomer and uses its GTPase activity to further contract the mitochondria, but it cannot complete the fission process due to the limitation of contraction strength (Frohlich et al., 2013). Dyn2/Dnm2 is a mitochondrial contraction and fission protein (Scholtes and Giguere, 2021) and, similar to Drp1, can form a cyclic oligomer around the membrane, further contracting the mitochondria and completing fission with the hydrolysis of GTP (Ferguson and De Camilli, 2012). In mammals, the two fission proteins must work in concert without one another.

In addition, the two GTPase proteins Dyn2/Dnm2 and Drp1 are depleted during mitochondrial fission, and when either protein is depleted, the mitochondria become elongated and display a finer tubular network. Mitochondrial fission begins when Drp1 is activated and is subsequently translocated from the cytoplasmic matrix to the OMM where it binds to the receptor, thereby initiating fission (Lee et al., 2016). However, the rate of fission depends on the amount of Drp1 binding and the level of phosphorylation modifications. For example, in neuronal cell, Ca^2+^ flux inward through voltage-dependent Ca^2+^ channels leads to a rapid arrest of mitochondrial motility and induces mitochondrial fission. The Ca^2+^ channels activate Ca^2+^/calmodulin-dependent protein kinase (CaMK)-alpha which in turn stimulates Drp1 serine (s600) phosphorylation, resulting in an increased affinity for Fis1 and promoting mitochondrial fission (Portz and Lee, 2021).

## Modulation of Mitochondrial Dynamics Under Pathophysiological Conditions

### Mitochondrial Fusion in Pathogenesis

During the transition from the G1/S phase in rat kidney cells, when large amounts of ATP are required by cells for nucleic acid and protein synthesis, the rate of mitochondrial fusion is significantly enhanced and the level of ATP in cells is much higher than during other periods (Mitra et al., 2009). However, there are many factors that alter the mitochondrial membrane potential, such as aging, apoptosis, and disease. Thus, whether there is a positive correlation between the mitochondrial fusion rate and membrane potential needs further investigation. When mice were exposed to high-fat diets, Mfn1 and Mfn2 expression was significantly decreased, accompanied by mitochondrial respiratory dysfunction and decreased skeletal muscle ATP levels (Liu et al., 2014). After swimming training in obese mice, ER stress was initiated and mitochondria-associated membrane content was increased, which in turn led to enhanced mitochondrial function and increased skeletal muscle ATP (Zhang et al., 2020). In severely starved mice, the rate of mitochondrial fusion and the mitochondrial network were significantly increased compared to well-fed mice; this resulted in elevated cellular AMP levels and activation of protein kinase A (PKA). In turn, PKA phosphorylated Drp1, resulting in a slowing of the mitochondrial fission rate, elevated fusion rate and OxPhos levels, and high expression of ATP synthase, maintaining the cellular ATP supply (Gomes et al., 2011). While starvation and stress can lead to the generation of more energy to maintain cellular function, they also increase the risk of mitochondrial reactive oxygen species (ROS) production and oxidative damage (Lee et al., 2014). Experiments using UV light and actinomycin D stimulation of mouse fibroblasts found that with enhanced mitochondrial fusion, ATP content and OxPhos levels were elevated and ROS levels were significantly elevated with increased mitochondrial damage and diminished function (Tondera et al., 2009). From the above, it is clear that there is a linear relationship between mitochondrial function and fusion rate, i.e., the rate of mitochondrial fusion is strongest when the fusion rate reaches some critical point, but at the same time, the risk of oxidative damage is greatest (Farmer et al., 2018).

### Mitochondrial Fission in Pathogenesis

In fibroblast studies, ionizing radiation triggered CaM-K alpha and activated Drp1 (s616) phosphorylation, thus accelerating the rate of lysis. However, when CaM-K alpha activity was inhibited, both Drp1 (s616) phosphorylation and lysis rates were significantly suppressed (Salaciak et al., 2021). Meanwhile, PKA mediated mouse fibroblast Drp1 (s637) phosphorylation allowing it to interact with Mff and be recruited to the OMM, resulting in an accelerated rate of mitochondrial fission (Yu et al., 2019b). Similarly, differences in nutritional status affect the rate of mitochondrial fission and ATP content in cells. Islet β-cells under high glucose and high fat nutritional conditions exhibit an increased mitochondrial fission rate, decreased FIS1 expression, reduced fusion rate, and reduced ROS production, triggering a mechanism that may be protective of normal cellular function by reducing excessive cellular ATP consumption (Molina et al., 2009). In skeletal muscle cells of high-fat dietary mice, Fis1 and Drp1 expression increased and fusion protein Mfn 1/2 expression decreased compared with control mice (Liu et al., 2014). Taken together, it is clear that when the organism (cell) contains high lipid levels, the rate of mitochondrial ATP synthesis is accelerated, which is also accompanied by an increase in ROS synthesis, increasing the risk of mitochondrial oxidative damage. Mitochondrial fission also removes damaged mitochondria, and healthy mtDNA and active substances are passed to the offspring mitochondria.

## Changes in Mitochondrial Dynamics Related Diseases

### Mitochondrial Dynamics and Neurodegenerative Diseases

Mitochondrial hyper fission leads to mitochondrial fragmentation and damage, decreased mitochondrial membrane potential, increased permeability, and decreased ATP production. At the same time, the disruption of mitochondrial autophagy leads to an excessive accumulation of intracellular mitochondria with abnormal function and high levels of oxygen radical production, producing large amounts of neurotoxic substances and eventually causing neurodegeneration disease (Portz and Lee, 2021; Yang et al., 2021). Parkinson’s disease, Alzheimer’s disease, and Huntington’s chorea, several neurodegenerative diseases are associated with mitochondrial dysfunction (Stanga et al., 2020; Table 2). Mendelian genetics seems to play an important role in the link between mitochondrial dysfunction and Parkinson’s disease (Buneeva et al., 2020). All three gene products responsible for familial autosomal recessive Parkinson’s disease can be found in mitochondria, including the Pink1 protein (a protein that maintains free radical metabolism, calcium homeostasis, and mtDNA in mitochondria) (Schapira et al., 2009; Schapira, 2012). It is also highly likely that Alzheimer’s disease is associated with polymorphisms in the OMM protein Tomm40 gene (Devi et al., 2006), which may be associated with age as an important risk factor for triggering Alzheimer’s disease (Roses et al., 2010). Similarly, Huntington’s chorea is associated with abnormal calcium handling due to mitochondrial defects, increased calcium-induced sensitivity to opening of mitochondrial permeability pores, and reduced mitochondrial respiration (Lim et al., 2008). Mutant Huntington proteins bind to the mitochondrial membrane, impairing axonal transport in mitochondria and reducing the synaptic ATP concentration (Orr et al., 2008). Mutant Huntington proteins also interact with and increase the sensitivity of inositol 1,4,5-trisphosphate receptors on mitochondria-associated membranes, thereby promoting calcium dysregulation in Huntington’s disease (Kaltenbach et al., 2007).

**TABLE 2:** Common diseases related to mitochondria dynamics.

**Diseases**	**Related genes and proteins**	**Mitochondria impact**	**References**
Alzheimer’s	APP, Presenilin	Fusion ↓ Fission ↑	Stanga et al., 2020
Parkinson’s	*Pink1, Parkin, VPS35*	Fission ↑	Tondera et al., 2009; Toda et al., 2016; Tezze et al., 2017
Charcot-Marie-Tooth disease type 2A	*Mfn2*	Fusion ↓	Twig et al., 2008; Touvier et al., 2015
Autosomal dominant optic atrophy	*Opa1*	Fusion ↓	Wai and Langer, 2016
Cardiomyocyte hypertrophy	*Drp1*	Fission ↑	Wang et al., 2012
Type 2 diabetes mellitus	*Mfn2*	Fusion ↓	Waterham et al., 2007; White et al., 2012

### Mitochondrial Dynamics and Diabetes

Mitochondrial DNA damage and malfunction are closely related to OxPhos and further affect oxidative stress. Diabetes associated with mitochondrial DNA abnormalities accounts for up to 1% of all cases of diabetes (Murphy et al., 2008), referred to as “mitochondrial diabetes.” Glucose induces neuronal activation and reduces ROS production in the ventral medial nucleus of the hypothalamus owing to the key role of uncoupling protein 2 (UCP2) (Toda et al., 2016), which regulates neuronal activity by controlling ROS production (Coppola et al., 2007; Andrews et al., 2008; Diano and Horvath, 2012). Furthermore, mitochondria in the ventral medial nucleus of the hypothalamus depend on UCP2 for regulation of the fission process (Toda et al., 2016). In summary, blood glucose concentration can regulate UCP2 secretion in the ventral medial nucleus of the hypothalamus, which in turn affects mitochondrial fission.

An imbalance in mitochondrial dynamics can cause pancreatic β-cell dysfunction and insulin resistance, thus inducing diabetes. An imbalance leads to a decrease in the level of mitochondrial OxPhos in the cell, proton efflux, mitochondrial membrane potential, and an increase in the production of ROS, which puts the cell in a state of oxidative stress. The inflammatory effect of the stress response reduces the sensitivity of the insulin signaling pathway (Gonzalez-Franquesa and Patti, 2017). First the expression level of Mfn2 affects the insulin signaling pathway (Mahdaviani et al., 2017); individuals with low Mfn2 expression in the liver are more likely to develop insulin resistance (Sebastian et al., 2012). Second, the expression level of Drp1 affects insulin resistance, and the mitochondrial Drp1 content is significantly increased in a mouse model of hyperinsulinemia. Inhibition of mitochondrial fission has been shown to improve obesity-induced insulin resistance in skeletal muscle. Myocardial biopsies from diabetic or hypoglycemic patients showed significantly higher mitochondrial deletion in myocytes than that in non-diabetic patients, and the number of deletions was higher and the sizes differed. Examination of skeletal muscle in diabetic patients revealed reduced levels of mitochondrial OxPhos (Kim et al., 2011). Electron microscopic observation of mitochondrial morphology in pancreatic islet β-cells of diabetic and obese patients revealed abnormal mitochondrial morphology, including fragmentation of mitochondria, destruction of mitochondrial cristae morphology, and a significantly lower volume and number of mitochondria than that of normal subjects. This is a more intuitive representation of the imbalance of mitochondrial fusion in the insulin-resistant state (Gonzalez-Franquesa and Patti, 2017).

This may be due to mitochondrial fragmentation caused by increased mitochondrial fission or decreased fusion. Therefore, mitochondrial dynamics are closely associated with the development of diabetes. It is hypothesized that an imbalance in mitochondrial dynamics under any circumstances can lead to β-cell dysfunction, manifested by decreased insulin secretion, β-cell failure, and eventually β-cell apoptosis, inducing the development of diabetes mellitus. One of the effective ways to improve the function of pancreatic β-cells is through regulation of Mfn2, Opa1, and other related mitochondrial dynamics proteins to maintain the balance of mitochondrial dynamics.

### Mitochondrial Dynamics and the Cardiovascular Diseases

When vascular endothelial cells (VEC) are stimulated by oxidative stress, their mitochondrial morphology becomes abnormal, as evidenced by increases in mitochondrial fission, abnormal mitochondrial structure, mitochondrial ROS production, and free radicals, and a decrease in OxPhos levels; VEC dysfunction occurs. Interference with mitochondrial fission is associated with myocardial ischemia/reperfusion injury; in animal models, inhibition of Drp1 expression in cardiomyocytes reduced myocardial infarct size and thus myocardial protection (Sharp, 2015). A significant decrease in Mfn2 and Drp1 expression was reported in heart failure. By interfering with mitochondrial fusion- and fission-related proteins, mitochondrial dynamics are affected, which in turn disrupts the normal physiological function of the VEC and induces many cardiovascular diseases (Givvimani et al., 2014). In addition, Mfn2 prevents cardiovascular I/R damage by enhancing mitochondrial fusion and activating the AMPK/Sirt3 signaling pathway (Liu et al., 2020). Furthermore, inhibition of Fis1 or Drp1 expression prevents high-glucose-induced ROS generation and mitochondrial fragmentation in venous endothelial cells with elevated levels of Fis1 protein in patients with atherosclerosis and increased abundance of Fis1 and Drp1 proteins in human aortic endothelial cells cultured in high-glucose medium (Mai et al., 2010).

### Mitochondrial Dynamics and Inflammatory Diseases

Mitochondrial dynamics imbalance leads to increased production of ROS in mitochondria, which has the effect of inducing Drp1 activation and fragmentation of mitochondria; the addition of ROS scavengers can prevent this process. Recently, it was found that the addition of mitochondrial division inhibitor 1 (Mdivi-1) or the knockdown of Drp1 in microglia inhibited ROS production (Maimaitijiang et al., 2016). In addition, ROS can lead to the release of Opa1 isoforms and cytochrome C from mitochondria into the cytoplasm, reduce apoptosis, and increase mitochondrial and cellular morphological stability (McBride and Soubannier, 2010; Huttemann et al., 2011). Interleukin-6 reduces mitochondrial Mfn2 protein expression by upregulating Fis1 expression and downregulating PGC-1α, biasing the mitochondrial dynamics balance toward fission (White et al., 2012). In the presence of tumor necrosis factor α (TNF-α), adipocytes 3T3-L1 exhibit abnormal mitochondrial morphology, increased Mfn2, and significantly increased Drp1 (Chen et al., 2010). However, TNF-α activates nuclear factor-κB (NF-κB) in pancreatic β-cells, and the activated NF-κB increases Opa1 expression, promotes mitochondrial fusion, and increases the level of mitochondrial OsPhos and ATP production, meeting the energy demand of mitochondrial fusion (Chan et al., 2012; Baltrusch, 2016). To avoid a critical reduction in total mitochondria, the TNF-α/NF-κB/Opa1 pathway is necessary to rebalance the system by increasing fusion proteins and enhancing the structure of mitochondrial ridges and the efficiency of the respiratory chain.

### Mitochondrial Dynamics and Muscle Diseases

Previous studies have demonstrated the important role of mitochondrial dynamics in maintaining muscle and mitochondrial physiological functions. Mice deficient in both mitochondrial fusion proteins Mfn-1, -2 in skeletal muscle exhibit severe mitochondrial dysfunction, mitochondrial DNA damage and severe developmental defects, which in turn affect a significant reduction in motor performance (Bell et al., 2019). In addition, Opa1 deficiency leads to mitochondrial kinetic imbalance, oxidative stress and inflammation (Tezze et al., 2017; Rodriguez-Nuevo et al., 2018), and Opa1 deficiency also promotes the secretion of FGF21 in skeletal muscle, leading to altered lipid homeostasis, inflammation and aging of different tissues (Tezze et al., 2017). Furthermore, inhibition of mitochondrial fission by gene silencing of Fis1 and Drp1 in skeletal muscle has been shown to reduce muscle atrophy caused by overexpression of transcription factor FoxO3a (Romanello et al., 2010). Interestingly, both low and high Drp1 levels impair skeletal muscle growth and development, and Drp1 overexpression late in life triggers mild muscle atrophy and decreased mitochondrial mass (Touvier et al., 2015; Giovarelli et al., 2020; Leduc-Gaudet et al., 2021). This also suggests that abnormalities in mitochondrial fission are also a factor in muscle disease.

## Therapeutic Potential of Targeting Mitochondrial Dynamics

Mitochondrial diseases are challenging to diagnose and treat clinically because of the high genetic and clinical heterogeneity of the disease. Previous preclinical studies have reported that benzofibrate improves mitochondrial function in Drp1-deficient cells with abnormal mitochondrial fission and function (Douiev et al., 2020). Benzofibrate is a small molecule activator of peroxisome proliferator-activated receptor alpha (PPARα), which upon activation increases the expression of many transcriptional regulators and thus affects the expression of downstream groups (Djouadi and Bastin, 2019). Genetic abnormalities in mitochondrial dynamics also include mutations in the Mfn2 or Opa1 genes, manifesting as Charcot-Marie-Tooth type 2A and autosomal dominant optic atrophy, respectively (Alexander et al., 2000; Zuchner et al., 2004), as well as by affecting Drp1 and Mff (Waterham et al., 2007). Therefore, specific inhibitors affecting mitochondrial fusion and fission have been studied to improve the pathophysiology (Cassidy-Stone et al., 2008; Wang et al., 2012; Qi et al., 2013). In contrast, P110, a peptide inhibitor with reduced Drp1 enzyme activity, can prevent Drp1/Fis1 interaction between neurons, which can effectively improve mitochondrial morphology and reduce mitochondrial fission, thus reducing neuronal axon loss in Parkinson’s disease patients and thus acting as a neuroprotective agent (Qi et al., 2013). This effect may be related to the reduction of apoptotic signaling pathway in neuronal cells mediated by Drp1-dependent p53 mitochondrial translocation. S3 acts on the mitochondrial deubiquitinating enzyme USP30, which regulates mitochondrial morphology by modulating Mfn1 and Mfn2 deubiquitination, and this action increases Mfn1 and Mfn2 activity and induces mitochondrial fusion, suggesting its potential therapeutic value for diseases such as insulin resistance (Yue et al., 2014). In addition, resveratrol, mitochondria-targeted antioxidants, and caloric restriction may improve insulin sensitivity and mitochondrial function in individuals with type 2 diabetes (Cao et al., 2018). Metformin and resveratrol protect mitochondrial integrity by inhibiting Drp1 activity and protect cellular function in hyperglycemic conditions (Maresca et al., 2013). Mitochondria-targeted antioxidants such as SS-31 protect normal cellular function in hyperglycemic environments by regulating mitochondrial membrane potential, inhibiting NADPH oxidase-4 and transforming growth factor-β1 expression, and activating p38MAPK. In addition, heat shock protein 70 can improve mitochondrial bioenergetic metabolism and reverse diabetic sensory neuropathy. All of the above drugs could be potential agents for the treatment of diabetic neuropathies, among others.

While fragmented mitochondrial networks are a component of disease pathophysiology, increasing mitochondrial fusion or reducing mitochondrial fission are both enhancements to the mitochondrial network in these cells and are expected to prevent functional deterioration. However, genetic and chemical approaches implemented in model systems that rebalance mitochondrial kinetics, restored mitochondrial structure and function alleviate disease-related symptoms. Research focused on dissecting the mechanisms of mitochondrial fusion and division and understanding the integration of these processes with other cellular pathways will be an important aspect of developing effective therapies, but enhancing the regulation of mitochondrial transport to improve cellular function in disease is a different idea and therapeutic strategy.

## Conclusion

A variety of factors and physiological and biochemical processes affect the degree of mitochondrial fusion or fission by regulating relevant factors that influence morphological changes in mitochondria, resulting in mitochondria showing different morphological characteristics. For example, increasing the degree of mitochondrial fusion or decreasing the degree of mitochondrial fission leads to the formation of larger, but fewer mitochondria in the cell. Conversely, decreasing the degree of mitochondrial fusion or increasing the degree of mitochondrial fission leads to the formation of smaller, but a large number of mitochondria. Thus, it can be speculated that when a disease or factor is associated with morphological changes in mitochondria, the process that is influenced can be identified. In summary, the role of mitochondrial fusion and fission proteins in the morphological structure and function of mitochondria has been elucidated. However, how breakthroughs in understanding mitochondrial dynamics can be better applied to existing research studies in the intervention of cell growth, aging, apoptosis, and other physiological and pathological processes needs to be studied more deeply.

## Author Contributions

Y-HC and C-JL: writing—original draft preparation. S-CL, C-HK, and C-JL: writing—review and editing. C-JL: supervision and funding acquisition. All authors have read and agreed to the published version of the manuscript.

## Conflict of Interest

The authors declare that the research was conducted in the absence of any commercial or financial relationships that could be construed as a potential conflict of interest.

## Publisher’s Note

All claims expressed in this article are solely those of the authors and do not necessarily represent those of their affiliated organizations, or those of the publisher, the editors and the reviewers. Any product that may be evaluated in this article, or claim that may be made by its manufacturer, is not guaranteed or endorsed by the publisher.
